# Screening of adolescent idiopathic scoliosis using generative adversarial network (GAN) inversion method in chest radiographs

**DOI:** 10.1371/journal.pone.0285489

**Published:** 2023-05-22

**Authors:** Jun Soo Lee, Keewon Shin, Seung Min Ryu, Seong Gyu Jegal, Woojin Lee, Min A. Yoon, Gil-Sun Hong, Sanghyun Paik, Namkug Kim

**Affiliations:** 1 Department of Industrial Engineering, Seoul National University, Seoul, Korea; 2 Department of Biomedical Engineering, Asan Medical Institute of Convergence Science and Technology, Asan Medical Center, University of Ulsan College of Medicine, Seoul, Republic of Korea; 3 Department of Orthopedic Surgery, University of Ulsan College of Medicine, Asan Medical Center, Seoul, Korea; 4 Department of Radiology, Hanyang University Hospital, Seoul, Korea; 5 Department of Radiology, Asan Medical Center, University of Ulsan College of Medicine, Seoul, Korea; 6 Department of Convergence Medicine, Asan Medical Institute of Convergence Science and Technology, Asan Medical Center, University of Ulsan College of Medicine, Seoul, Republic of Korea; Sejong University, REPUBLIC OF KOREA

## Abstract

**Objective:**

Conventional computer-aided diagnosis using convolutional neural networks (CNN) has limitations in detecting sensitive changes and determining accurate decision boundaries in spectral and structural diseases such as scoliosis. We devised a new method to detect and diagnose adolescent idiopathic scoliosis in chest X-rays (CXRs) employing the latent space’s discriminative ability in the generative adversarial network (GAN) and a simple multi-layer perceptron (MLP) to screen adolescent idiopathic scoliosis CXRs.

**Materials and methods:**

Our model was trained and validated in a two-step manner. First, we trained a GAN using CXRs with various scoliosis severities and utilized the trained network as a feature extractor using the GAN inversion method. Second, we classified each vector from the latent space using a simple MLP.

**Results:**

The 2-layer MLP exhibited the best classification in the ablation study. With this model, the area under the receiver operating characteristic (AUROC) curves were 0.850 in the internal and 0.847 in the external datasets. Furthermore, when the sensitivity was fixed at 0.9, the model’s specificity was 0.697 in the internal and 0.646 in the external datasets.

**Conclusion:**

We developed a classifier for Adolescent idiopathic scoliosis (AIS) through generative representation learning. Our model shows good AUROC under screening chest radiographs in both the internal and external datasets. Our model has learned the spectral severity of AIS, enabling it to generate normal images even when trained solely on scoliosis radiographs.

## Introduction

Adolescent idiopathic scoliosis (AIS) is the most common spinal deformity [1] and is defined as a 10° or more spinal curvature of unknown etiology in persons 10 to 18 years old [2]. AIS has an overall 0.47% to 5.2% prevalence [3]; thus, screening in school-aged adolescents is imperative because early detection can reduce the need for surgery through non-surgical management such as bracing [4]. However, Cobb’s angle measurements conveyed a 4° to 8° intra-and inter-observer variability [5, 6], with one study revealing a potential maximum inter-observer measurement error up to 11.8° [7]. In addition, Cobb’s angle manual measurement is labor-intensive and time-consuming [8]. Therefore, it would be conducive to authentic clinical practice if Cobb’s angle was only measured in suspected scoliosis patients, which can be completed during health checkups.

Thus, many studies actively research AIS diagnosis using deep learning models with convolutional neural networks (CNN). For example, one study developed an automated scoliosis screening algorithm using a deep learning model for naked back photos [2]. Furthermore, as several segmentation studies incorporate deep learning [9], some authors have developed an automated Cobb’s angle measurement algorithm using the detection or segmentation provided by another deep learning model [10–12]. However, supervised learning will inevitably function poorly with external dataset images. Moreover, CNN is affected by image textures [13], so it may not be preferable for diagnosing spectrum disorders that affect the global spinal structure, such as scoliosis. Therefore, a method that can withstand continuous or spectral progression would complement the clinical field’s discriminative diagnosis tendency, which inevitably binarizes patients with borderline symptoms into the ‘normal’ category.

In this regard, we noticed an alternative machine learning field trend: finding a lower dimensional data representation with preferable properties such as discernibility through data learning distribution. Specifically, we developed a deep learning AIS diagnosis model with classification features that are latent vectors acquired from query images extracted using GAN inversion. In this feature-extracting process, we incorporated GAN trained from an imbalanced dataset that does not include standard counterpart samples to maximize the differentiating ability. Therefore, we conducted an ablation study to find and evaluate a model that exhibits optimal performance and diagnostic power. The primary purposes of the study are:

- Empirically proving GAN’s ability to generate normal images through partial normality, provided in training, set with symptoms.
- Developing a novel method for differentiating given data using GAN as a feature extractor.

Our method is expected to detect spectral disease progressions with high sensitivity.

## Literature review

Representation learning is a machine learning method allowing a system to learn feature identification from a substantial amount of unlabeled data [14–16]. While most works learn data distribution representation using a discriminative objective, some studies have utilized a generative learning approach that generates or models pixels in the input space [17–19]. Multiple investigations have attempted to interpret the relationship between data and its latent representations in generative models. Arora et al. [20] proposed that using a generative adversarial network (GAN) as a feature learner is a practical approach due to its low support in GANs-learned distribution; they also provided a theoretical background for this approach. Salimans et al. [21] proposed semi-supervised learning through discriminator modification to classify K-classes and a virtual class for detecting fake samples. On the other hand, Srinivasu et al. [22] injected information from the generative model in tackling discriminative tasks by using autogenerated images reconstructed using variational autoencoders [18] as additional data for model training. He et al. proposed a novel unsupervised learning approach using masked autoencoders to reconstruct inputs from partially masked versions of themselves [23]. This allows the model to learn suitable representations for downstream tasks, particularly in computer vision applications. While their approach is purely unsupervised in the upstream phase, our proposed method leverages semi-supervised learning on a dataset with abnormal labels, making it somewhat similar to He et al.’s approach.

These studies displayed the data distribution and learned latent space correlation, thus suggesting a similar correlation in a specific data sample-latent vector pair. However, the abovementioned methods are limited as they require extra architectural modules or modifications. Thus, methods that manipulate the latent vector were introduced. StyleGAN [24] introduced intermediate latent space W, which is more disentangled semantically, further boosting latent vector investigations. SeFa [25] provided an unsupervised algorithm to identify dominant directions in the latent space.

The present study uses the GAN inversion method for generative representation learning on a relatively small-sized image dataset. GAN inversion discovers a code in the GAN-trained latent space, generating the best reconstruction of a given query image. The most direct and intuitive GAN inversion method is the optimization-based method proposed by Abdal et al. [26]. This optimization-based method tries to find *z*,* which satisfies the following Eq (1):

$$z^{*}={\text{argmin}}_{z}\;\ell (\text{G}(\text{z}),\;\text{x}),$$ (1)

where ℓ(image, image) is a predefined similarity metric, x is the given query image, and G(z) is the latent vector generated image. z* corresponds to the latent manifold vector that resembles the given query image the most. In this sense, we propose an alternative novel method utilizing features embedded in an intermediate manifold. Specifically, the vector acquired using the generative adversarial network (GAN) inversion [26] is equivalent to the discriminative method’ extracted feature as the inherent information about the given images is embedded in the acquired vector. This feature extraction differs from conventional models that utilize CNNs as CNN models craft features by accumulating information acquired from convolution operations on image patches. In contrast, our method extracts more general, large-scale features from the whole image.

## Materials and methods

### 1 Dataset and preparation

This retrospective study followed the Declaration of Helsinki principles [27]. The study protocol was approved by our institution’s (Center 1) Institutional Review Board Committees and other institutions (Center 2), which waived the need for written informed patient consent.

#### 1.1 Dataset

**Fig 1:**
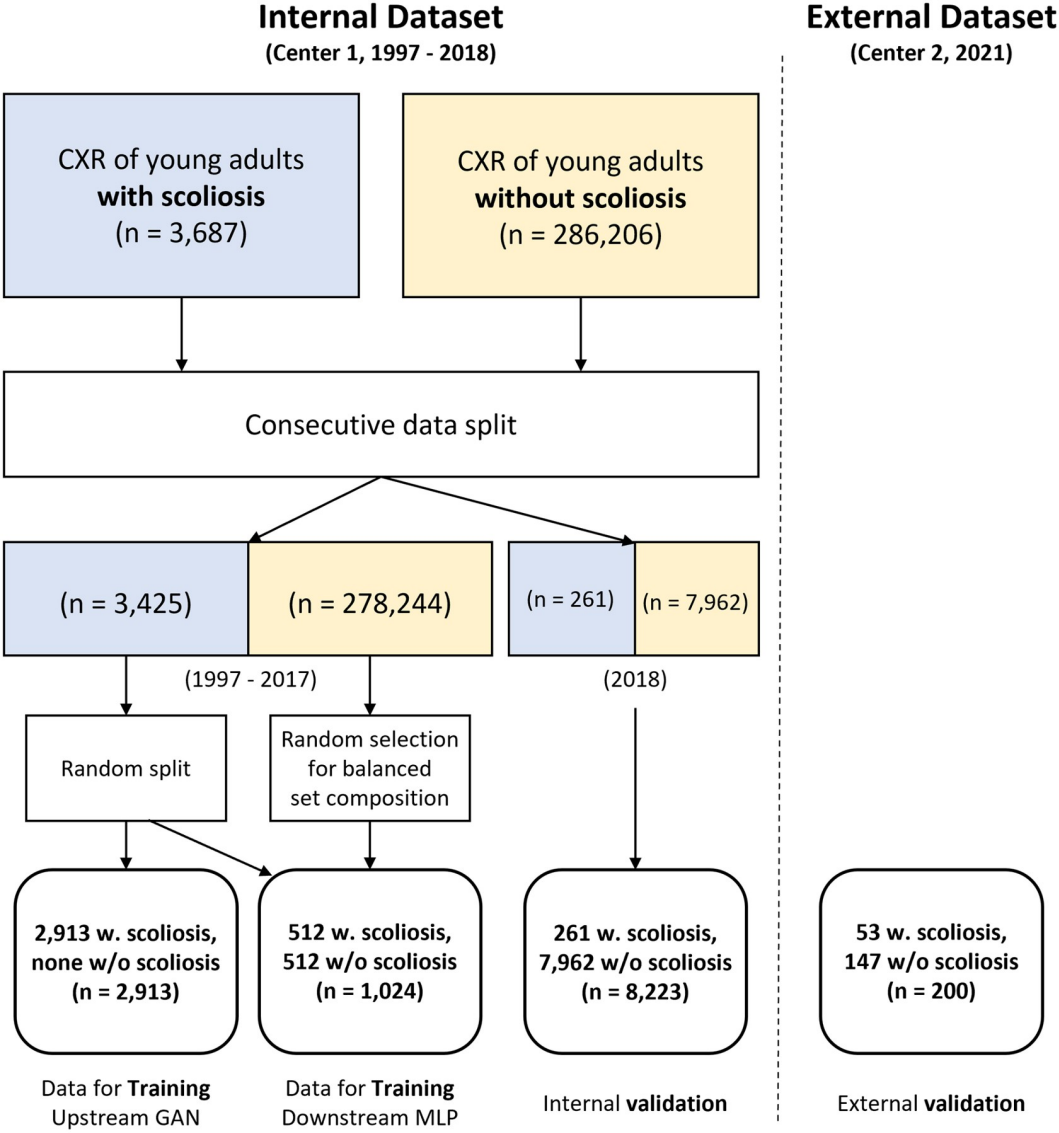
A schematic diagram of collected data.

Fig 1 depicts a data collection and split schematic. We chronologically split the training and validation datasets by using a portion of the data obtained between 1997 and 2017 as the training data and all data obtained in 2018 as the validation data. When composing the training dataset, all chest X-rays (CXRs) with AIS were used, and some normal counterpart CXRs were randomly selected to form a balanced dataset. As our model requires two-stage training, we randomly split the collected CXRs with AIS into two datasets: upstream GAN and downstream multi-layer perceptron (MLP). The diagnostic threshold was set as 20 degrees considering that close observation is generally recommended for patients with an initial Cobb’s angle of fewer than 20 degrees [28]. An orthopedic surgeon with eight years of clinical experience did the angle measurement. The exclusion criteria were cases post-spine surgery or of younger children. As a result, we obtained three datasets.

The first dataset collected 2,913 CXRs of young adults with scoliosis to train the upstream GAN, which was used for feature extraction. The second dataset comprised 1,024 CXRs of young adults collected at Center 1 with a 1:1 normal-to-scoliosis ratio for training the downstream MLP to detect scoliosis. The final dataset constituted 8,223 CXRs of young adults collected at Center 1; 261 had scoliosis, and 7,962 had no noticeable disease. For external validation, we collected 53 CXRs with scoliosis and 147 CXRs without scoliosis from Center 2 in 2021. In addition, after the study began, the most recent 200 cases were selected retrospectively and externally validated. More detailed demographic information about the collected dataset is provided in Table 1.

**Table 1:** Detailed demographic information of the collected datasets.

	Center 1			Center 2
	Upstream GAN	Downstream MLP	Internal validation	External validation
Radiograph quantity	2,913	1,024	8,223	200
Scoliosis	2,913	512	261	53
Normal	-	512	7,962	147
Age	14.40 ± 2.25	14.90 ± 2.26	14.40 ± 2.68	18.64 ± 4.17
Scoliosis	14.40 ± 2.25	14.45 ± 2.23	14.59 ± 2.01	22.42 ± 3.29
Normal	-	15.34 ± 2.21	14.39 ± 2.70	17.27 ± 3.58
Sex (M/F/O)	754/1473/686	354/407/263	4720/3481/22	69/131/0
Scoliosis	754/1473/686	135/262/115	63/197/1	10/43/0
Normal	-	219/145/148	4657/3284/21	59/88/0
Collection year(s)	1997–2017	1997–2017	2018	2021

M, Male; F, Female; O, Others (anonymized data). Age is presented as mean ± standard deviation.

#### 1.2 Image preprocessing

As our task focused on global skeletal orientation and not fine features on soft tissues, image preprocessing was to enhance the bone area and preserve the aspect ratio. First, we checked the image spacing information to preserve the image aspect ratio if the image was distorted due to different vertical/horizontal spacing ratios. Then, we applied CLAHE [29, 30], an equalizing histogram technique to improve contrast on image patches, with a 2.0 clipLimit and (8,8) tileGridSize on input images to differentiate vertebral bones from soft tissues, especially in the lumbar vertebral area. To introduce the CXR into the model, resizing to 512 × 512 resolution was required. However, naive resizing would change the aspect ratio, unintendedly altering Cobb’s angle, which is the gold standard for indicating scoliosis. Therefore, we added padding to the image so the original image’s aspect ratio would not change, even when resized. Next, we stacked the processed black-and-white image channel-wise to mirror the same shape as natural RGB images.

### 2 Methods

#### 2.1 Training GAN with the diseased dataset

**Fig 2:**
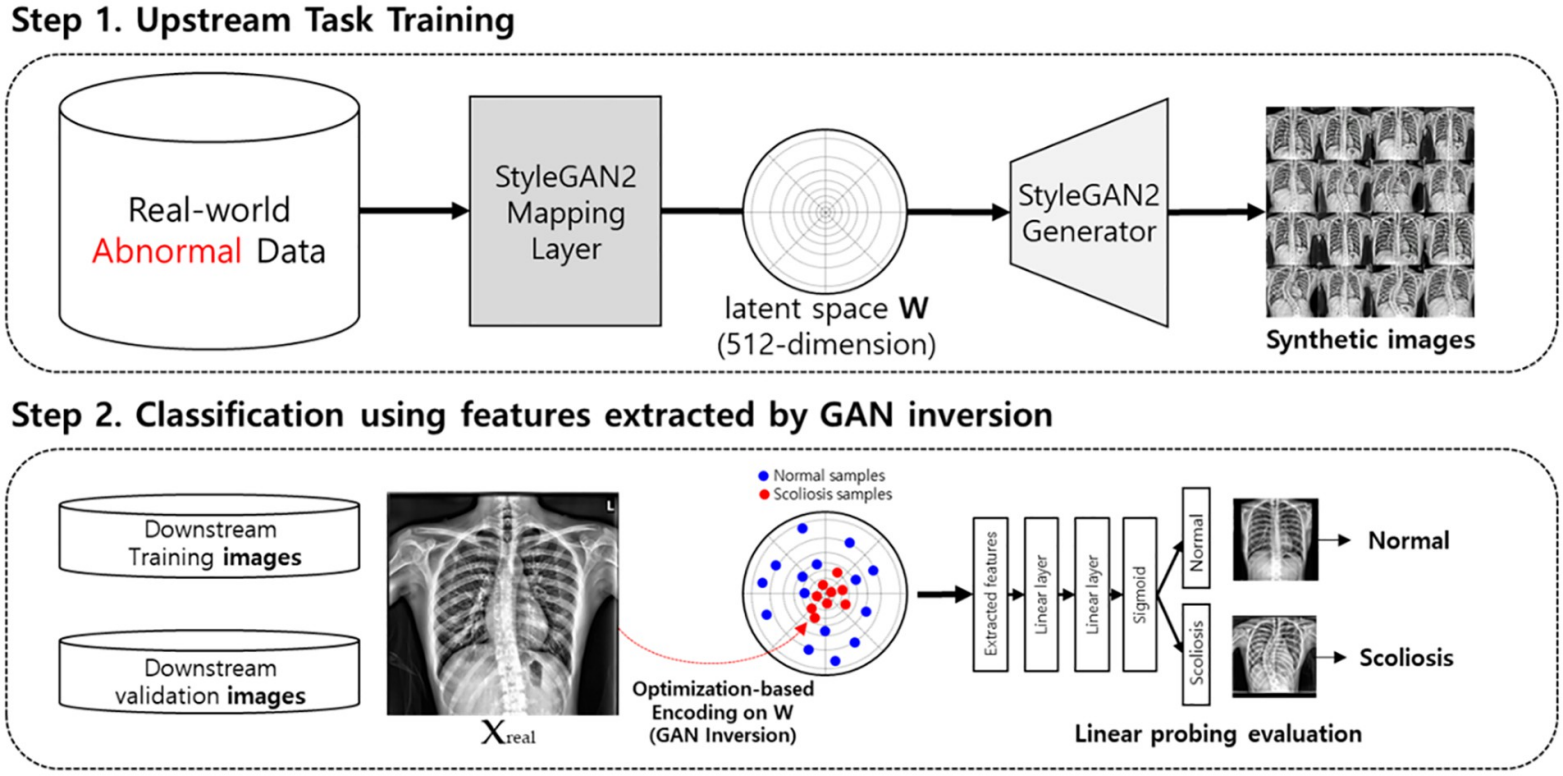
Training strategy for developing sensitive classification networks using GAN inversion as a feature-extracting method. The first step is an upstream task that trains the generative adversarial network (GAN). The second step classifies through linear probing and projection combination using GAN inversion.

Fig 2 illustrates our proposed method. The training dataset usually includes a natural distribution for maximum generated-image diversity for the training GAN. However, data scarcity results in low-quality images with minimal intra-class variation. Shahbazi et al. noted that this tendency depreciates the conditional training [31]. Considering our objective and dataset size, we trained our network using only CXRs expressing some scoliosis degree. Using the scoliosis classification criteria set by Goldstein [32] and Cruickshank [33], curve patterns were determined by observing where the curve apex exists. From this standard, we noted that even in severe scoliosis cases, some parts could be diagnosed as normal in focal view. For example, local thoracic spine observation could determine little difference in a CXR with severe scoliosis on the lumbar spine from the thoracic spine without scoliosis. Therefore, we hypothesized that we could more effectively embed the disease axis into the model feature space without degrading the normal distribution too much by using an imbalanced dataset. An empirical analysis justifying our method is provided in the discussion section.

#### 2.2 GAN inversion for feature extraction

We conducted optimization-based method experiments considering data scarcity and embedded our vector into the original W space instead of the extended W+ space. Karras et al. [24] and Yang et al. [34] established that different semantics are hierarchically determined from different resolution layers. Therefore, embedding vectors in a fine-grained manner would benefit fine feature reconstruction, which was not the primary goal of this study. Next, we suspected embedding into W+ space would bring excessive computational cost as it has a much higher dimension than W space.

#### 2.3 Evaluation

Supervised learning combined latent codes (through GAN inversion-extracted features) and scoliosis presence based on the original image. The projection head evaluated GAN’s latent space discriminative ability [35], similar to the widely used linear probing method [36] in self-supervised learning evaluations. The projection head is a 2-layer MLP; hidden MLP layers were 512 dimensions and used ReLU [37], and output MLP layers were one dimension with a sigmoid. None of the MLP layers contained batch normalization [38].

First, each performance was analyzed using a likelihood value threshold between 0 and 1 extracted by the MLP layers. We evaluated the 95% confidence interval (CI) and the area under the receiver operating characteristics curve (AUROC) to determine whether the model performance was significantly better. Since this study aimed to screen AIS in a real-world setting, we fixed the sensitivity at 0.9 and calculated the true-positive (TP), false-positive (FP), true-negative (TN), and false-negative (FN) values. In addition, the quantitative classification assessment included accuracy, sensitivity, specificity, positive predictive value (PPV), and negative predictive value (NPV). Finally, an orthopedic surgeon with eight years of clinical experience visually analyzed the FP/FN cases to evaluate the method’s performance. We calculated the accuracy, sensitivity, specificity, PPV, and NPV to evaluate the method’s efficiency in a clinical AIS diagnosis scenario. We compared performance by adjusting our model’s threshold relative to screening purposes.

Second, we conducted an ablation study to classify performance relative to MLP stack numbers. In addition, a data stress test was conducted to confirm classification performance relative to latent code numbers used for the downstream training phase.

Third, we investigated whether different pre-trained weights affected the feature extraction performance using the GAN inversion method by evaluating the downstream classification performances corresponding to different weights.

Fourth, while we hypothesized that normal images could be embedded well into the latent space formed by learning the abnormal image distribution, it is against AnoGAN’s widely-accepted philosophy [39]. Therefore, to determine that our trained latent space has expanded enough to generate normal images, we conducted qualitative and quantitative analyses on our approach’s ability to embed normal images.

#### 2.4 Training configurations

We utilized StyleGAN-ADA [40] architecture for training the upstream GAN network. We inherited implementation details concerning Pytorch [41] implementation without modifications from the study’s authors [40]. The training data was preprocessed following the method mentioned in *Section 3*.*1*.*2*. The input data augmentation to the discriminator during styleGAN2-ADA training was performed with maximal provided pipeline combinations. However, we excluded some augmentations not applicable to medical deep learning, such as horizontal flip or cutout. We used a non-saturating loss [19] with R1 regularization [42], utilizing 6.5536 as the coefficient value for the loss function. Finally, we used the ADAM [43] optimizer with a 0.002 learning rate. Next, we used the Frechet inception distance (FID) [44] on the total training dataset to evaluate the upstream network’s convergence. We selected epochs with the lowest FID value after training for 1 million iterations, so the FID conveys convergence.

We used a simple loss for feature extraction that minimizes the L2 norm of the given query and generated images with a noise regularization term. Then, we iterated 1000 times to extract the final vector without weight update to the GAN generator. For classifying the extracted vectors, we used binary cross-entropy as a loss to train the binary classifier. The downstream training set’s normal-to-abnormal ratio was set to 1:1, and the training data in each dataset started from 32 and doubled up to 1024. The model was trained for 200 epochs with complete batch learning, and the learning rate was set to 0.001 in the Adam optimizer.

## Results

### 1. Classification result

**Fig 3:**
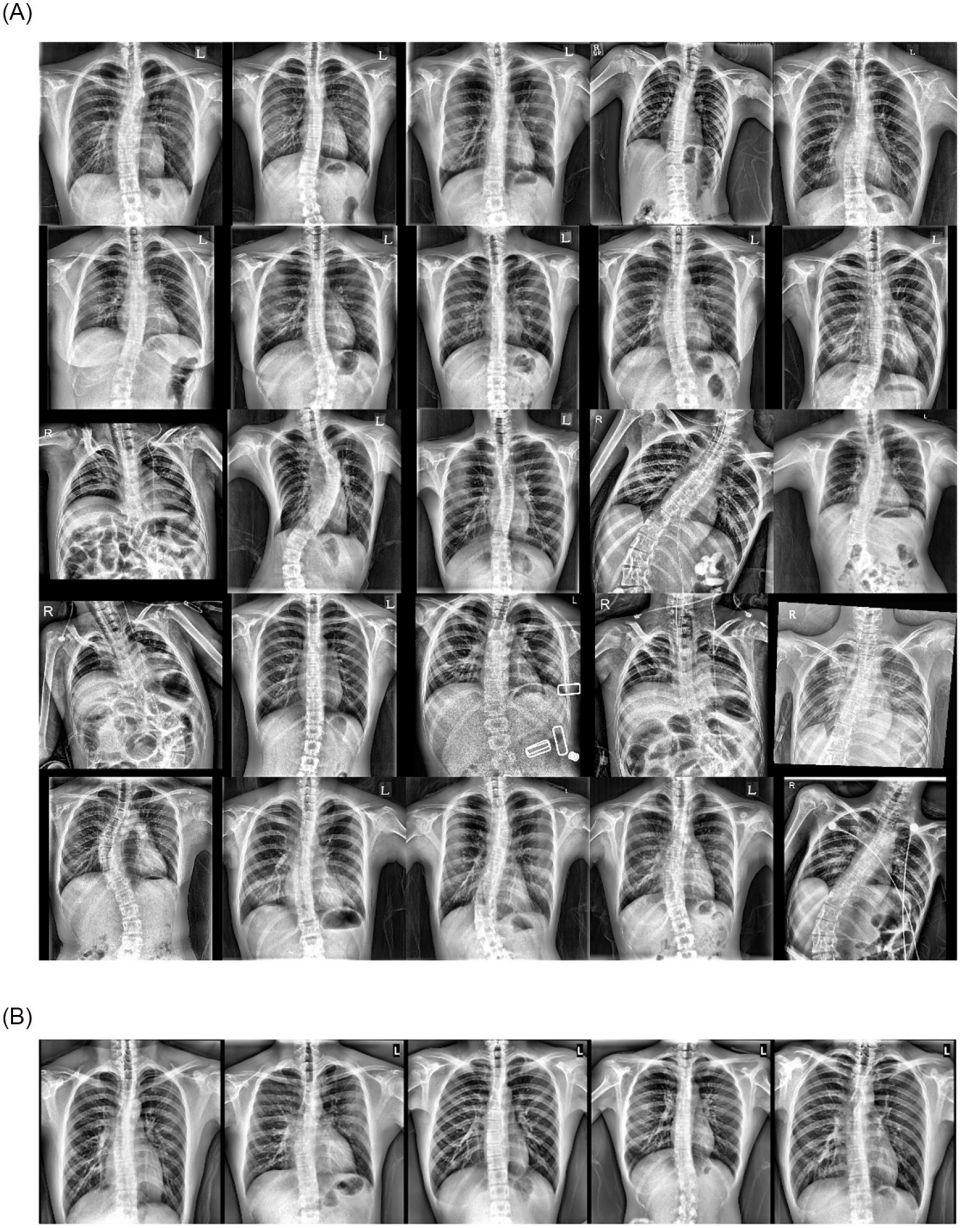
False-negative case examples in the (A) internal test and (B) external validation datasets.

The threshold was set at a 0.9 sensitivity in the screening setting, and our model’s internal and external validation results are organized in Table 2. When the sensitivity was set to 0.9 in the internal and external datasets, the specificity was 0.697 and 0.646, respectively. There were 25 FN cases of our model in the internal test dataset and 5 FN cases in the external test dataset, which are shown in Fig 3.

**Table 2:** Our final model’s scoliosis classification performance.

Test dataset	Performance measure					
	AUROC	ACC	SEN	SPE	PPV	NPV
**Internal**	0.850	0.704	0.9*	0.697	0.096	0.995
**External**	0.847	0.715		0.646	0.480	0.950

AUROC, area under the receiver operating characteristic curve; ACC, accuracy; SEN, sensitivity; SPE, specificity; PPV, positive predictive value; NPV, negative predictive value

### 2. Ablation study

**Fig 4:**
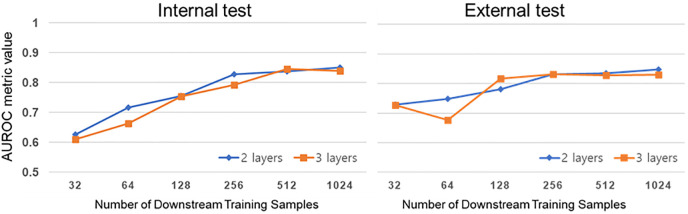
Scoliosis classification performance within the number of projection head layers and downstream training samples.

The AUROC evaluation results relative to the layer numbers in the projection head and downstream training samples are summarized in Fig 4. This evaluation incorporated a linear protocol to evaluate the classification performance. As sample quantities increased, the AUROC tended to improve. When the projection head layer number was 2 and the downstream training sample was 1024, internal and external AUROCs were 0.850 and 0.847, respectively, indicating the highest classification performance.

### 3. Pre-trained weight effect of the upstream task

**Fig 5:**
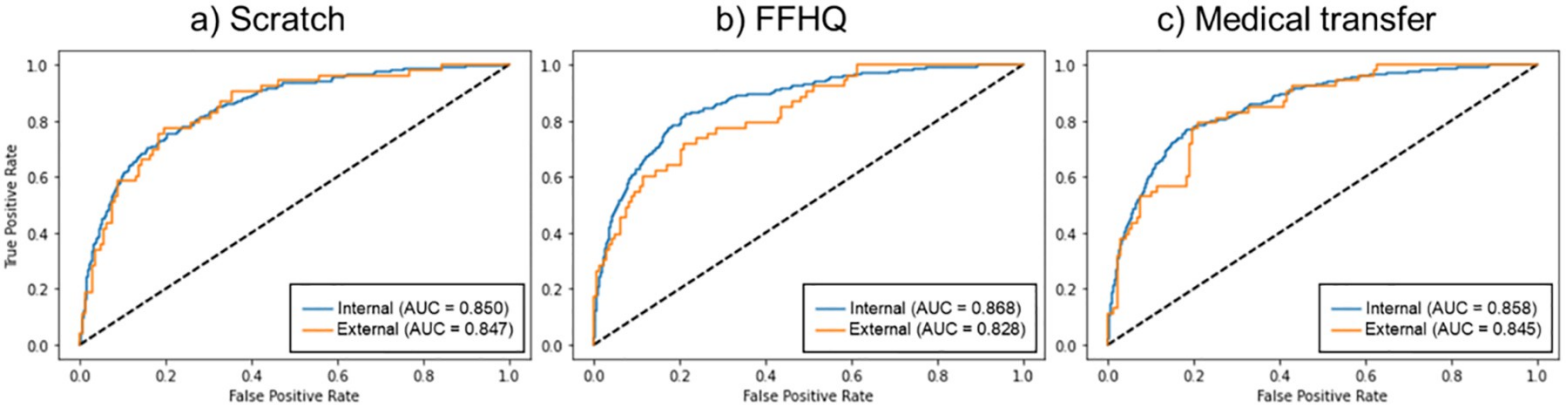
Downstream validations relative to upstream pre-trained weight type.

We evaluated performance relative to pre-trained weights, and the results are summarized in Fig 5 and Table 3. Internal and external AUROCs were 0.850 and 0.847 for scratch training, respectively; AUROCs with Flickr-Faces-HQ (FFHQ) [24] pre-training weights were 0.868 and 0.828, and medical transfers were 0.858 and 0.845, respectively. When considering scratch and FFHQ pre-trained weights, the downstream classification result differences were not statistically significant. For FFHQ, internal AUROC increased by 0.028 compared with scratch, whereas external AUROC decreased by 0.019. Lastly, scratch training and medical transfer pre-trained weights were used to analyze the downstream AUROC. Internal AUROC increased by 0.008, and external AUROC decreased by 0.002. Similarly, there was no statistically significant difference.

**Table 3:** Performance metric comparison under a fixed 0.9 sensitivity and statistical analysis using independent t-test among different feature extractor weights.

	**Scratch**		**FFHQ**		**Medical**	
	Internal	External	Internal	External	Internal	External
**95% CI**	0.842–0.858	0.789–0.894	0.833–0.849	0.768–0.878	0.825–0.841	0.787–0.892
**AUROC**	0.850	0.847	0.868	0.828	0.858	0.845
**Sensitivity (fixed)**	0.903	0.906	0.904	0.906	0.897	0.906
**Specificity**	0.585	0.646	0.581	0.503	0.580	0.585
**PPV**	0.066	0.480	0.066	0.397	0.065	0.440
**NPV**	0.995	0.950	0.995	0.937	0.994	0.945
Statistical analysis (*P* values of independent t-test)						
	**Scratch vs. FFHQ**		**Scratch vs. Medical**		**FFHQ vs. Medical**	
**Internal**	0.636		0.383		0.447	
**External**	0.534		0.950		0.588	

CI: confidence interval, AUROC: area under the receiver operating characteristic curve, PPV = positive predictive value. NPV = negative predictive value.

Scratch refers to training without pre-trained weight. FFHQ refers to training pre-trained weight using Flickr Faces HQ Dataset. Medical refers to training pre-trained weight using chest radiograph.

### 4. Quantitative analysis for method validation

**Fig 6:**
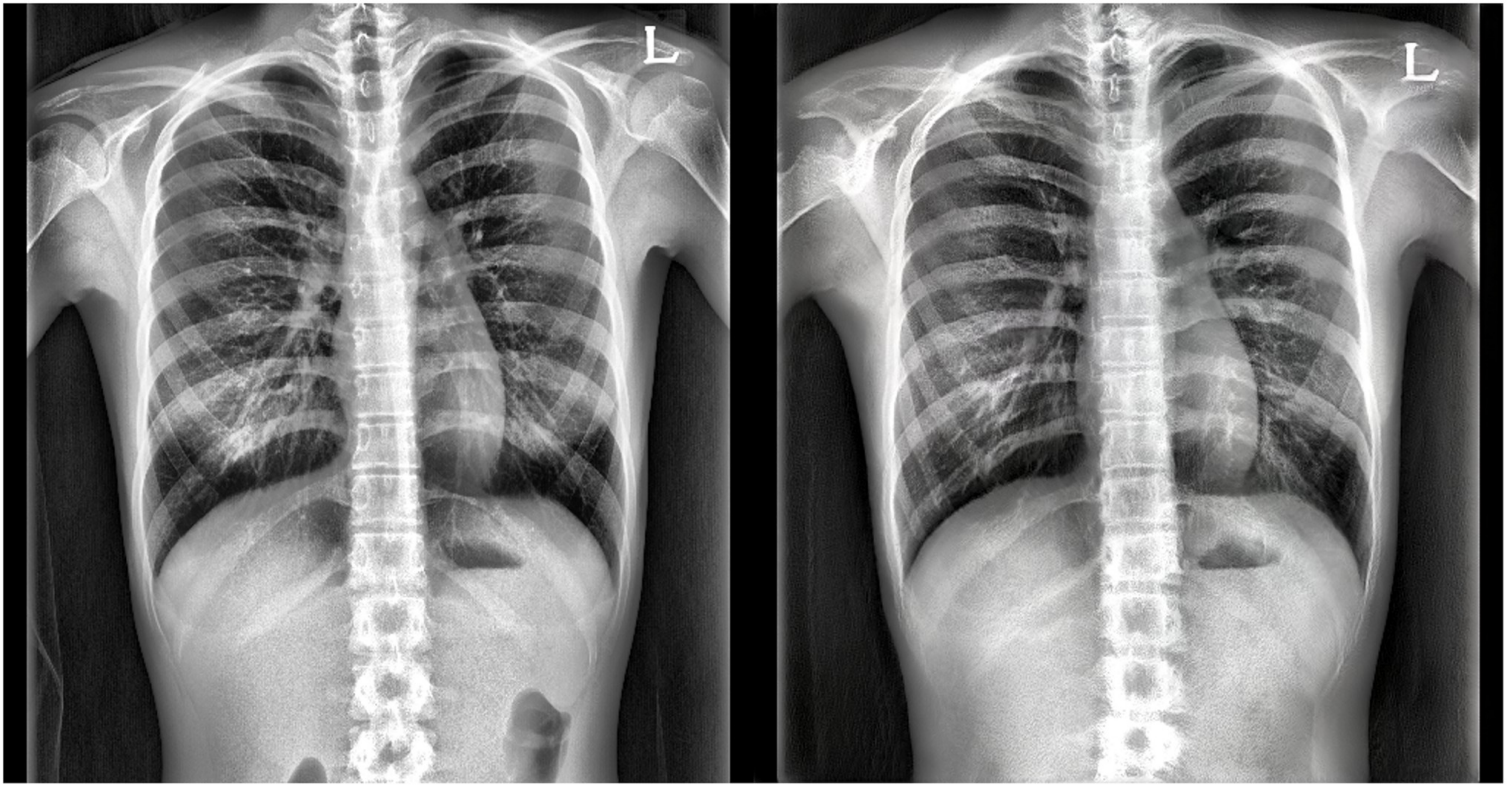
Example of a well-reconstructed normal sample. (Left) original chest X-ray. (Right) Reconstructed image using the GAN inversion technique. The encoding generator was only trained with chest X-ray images with scoliosis.

We calculated query and projected image peak signal-to-noise ratios (PSNR), structural similarity index measures (SSIM) [45], and root mean square errors (RMSE) to quantitatively compare how well images embedded into the latent space. Based on the ground truth, we calculated these three metrics on every sample in the downstream training set. Table 4 shows the average image-reconstruction quality metric values measured on scoliosis and the normal downstream training set. Images with scoliosis were better reconstructed according to SSIM and RMSE metrics, whereas images without scoliosis were better reconstructed with PSNR metrics. Finally, we selected a well-reconstructed normal image example using vectors from the GAN inversion method to further corroborate these results (Fig 6).

**Table 4:** Image-reconstruction quality metrics of scoliosis and normal downstream training sets.

Metrics	PSNR	SSIM	RMSE
Data			
**Scoliosis**	19.048 ± 2.110	0.464 ± 0.108*	8.659 ± 0.742*
**Normal**	19.398 ± 1.313*	0.432 ± 0.059	8.887 ± 0.505
^†^ ***P* value**	0.002	<0.001	<0.001

^†^Paired t-test for comparing image reconstruction quality metric among disease presence.

*superior data among scoliosis or normal dataset.

PSNR, peak signal to noise ratio; SSIM, structural similarity index measure; RMSE, root mean square error.

## Discussion

This study evaluated a novel deep learning model’s AIS diagnosing accuracy using latent vectors acquired from query images using GAN inversion as features. Based on the best performance in the dataset, internal and external dataset AUROCs were 0.850 and 0.847, respectively. Furthermore, we provide internal and external dataset ROC curves in Fig 5a. Our method indicated good generalizability because the AUROC value did not degrade drastically when tested on an external dataset. Therefore, our model is a potential tool in practical AIS screening. In the results depicted in Table 2, the sensitivity was fixed at 0.9 because this model’s primary purpose was for AIS screening. Despite the specificity result being inevitably lower than sensitivity, the internal and external dataset specificities were 0.697 and 0.646, respectively. Thus, we believe this model may be preferable for real-world use.

**Fig 7:**
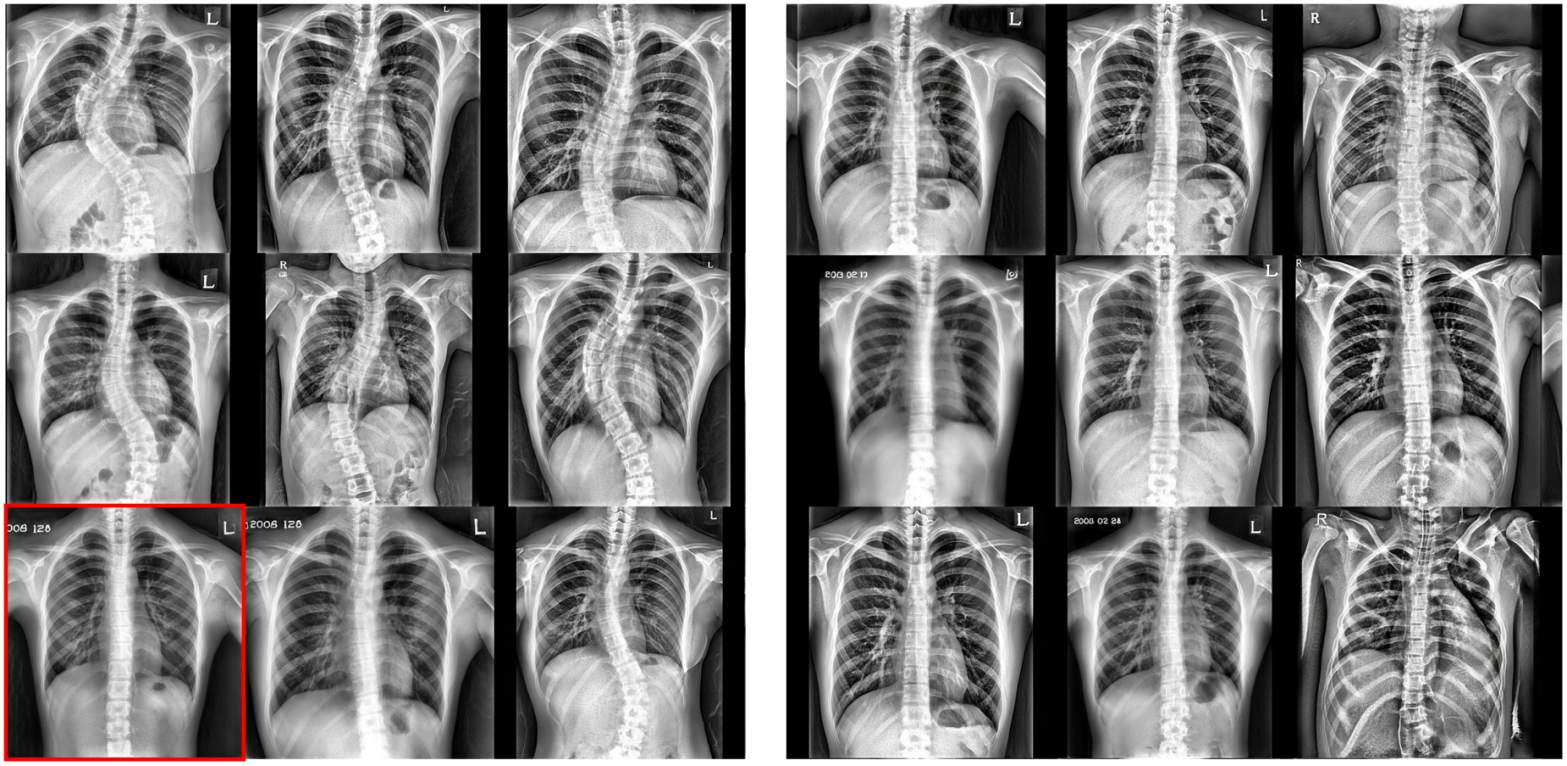
Generated CXR samples. (Left) Samples from a model trained on an imbalanced dataset (our method). (Right) Samples from a model trained on a balanced dataset. Red box: a sample radiograph that can be diagnosed as a normal spine in generated samples.

We used a toy comparison experiment using a balanced dataset to justify using an imbalanced dataset as the training dataset. Fig 7 illustrates the generated CXR images that only differ in the training dataset composition, indicating that samples generated from the model trained with an imbalanced dataset have much more diversity regarding scoliosis severity and location. Furthermore, a sample that can be diagnosed as normal (red box in Fig 7) was also included in generated samples, further supporting our hypothesis that the latent space can be expanded to generate normal samples even when trained on a scoliosis-only dataset. On the other hand, samples generated from the model trained on a balanced dataset express little to no diversity in scoliosis scope, where all generated samples have “straight” spines.

The internal 25 FN out of 261 scoliosis case validation analysis demonstrated that 36% of cases were levoscoliosis (Fig 3). In addition, an American Academy of Family Physicians review estimated that 10 to 15% of adolescents with scoliosis had left curves or levoscoliosis [46]. Therefore, the FN cases are assumed as incorrect because levoscoliosis radiographs were rarely included during training. Since levoscoliosis can be recognized as a negative Cobb’s angle from the SeFa factorization perspective [25], where we theorize dextroscoliosis progression as the prominent data distribution variation, they occupy a part of the supernormal, not both scoliosis and normal. However, it is postulated that these false cases occurred because dextroscoliosis and levoscoliosis were not distinguished.

We also checked FP cases and found that some external devices were visible in 1,838 of the 3,297 cases, including Hickman catheters, chemo ports, vital sign monitor lines, and cardiovascular devices, such as cardiac pacemakers or implantable cardioverter-defibrillators. We assume that tube curves were incorrectly precepted as a bent spine since we designed our model to be sensitive to structural changes. Therefore, we manually excluded all cases with external devices from the test dataset and calculated the metrics again. As a result, the AUROC increased from 0.850 to 0.894in the internal test dataset, and the specificity increased from 0.585 to 0.697 when the sensitivity was fixed at 0.9. However, these metrics’ significance could not be verified as data sample quantities differed.

According to Fig 4, the best classification performance was achieved when the projection head layers were two instead of three, and the downstream training sample quantity was 1024. Previous studies did not have a promised projection head structure [35, 47, 48]. However, Chen et al. conducted a data stress test with projection head layers ranging from two to four and noted that a larger layer quantity was associated with higher representation performance [49]. This trend seems stronger with a smaller downstream data volume, but the above characteristic did not appear as our downstream data set is a very small scale of only 1024 samples. From the data stress test results in Fig 4, when linear probing was performed with 256 labeled samples, the internal and external AUROCs expressed sufficiently high performance within a 0.828 and 0.831 data limitation, respectively. Although the labeled data amount was reduced by a quarter, the best-performing internal and external AUROC difference was only 0.022 and 0.016, a notable advantage of our method.

As for the ablation study represented by Table 3, we examined whether providing prior knowledge on training upstream GAN boosts model performance, specifically on training upstream GAN. We evaluated fine-tuning pre-trained weights effects [50] compared with upstream training networks from randomly initialized settings. We used FFHQ pre-trained weights [40] and a trained weight on a private 200,000 CXR images dataset from Center 1. Since FID demonstrated the best delegate diversity measure in the generated image set [44], better quality FID-generated images from fine-tuned GAN could not be applied to our task.

**Fig 8:**
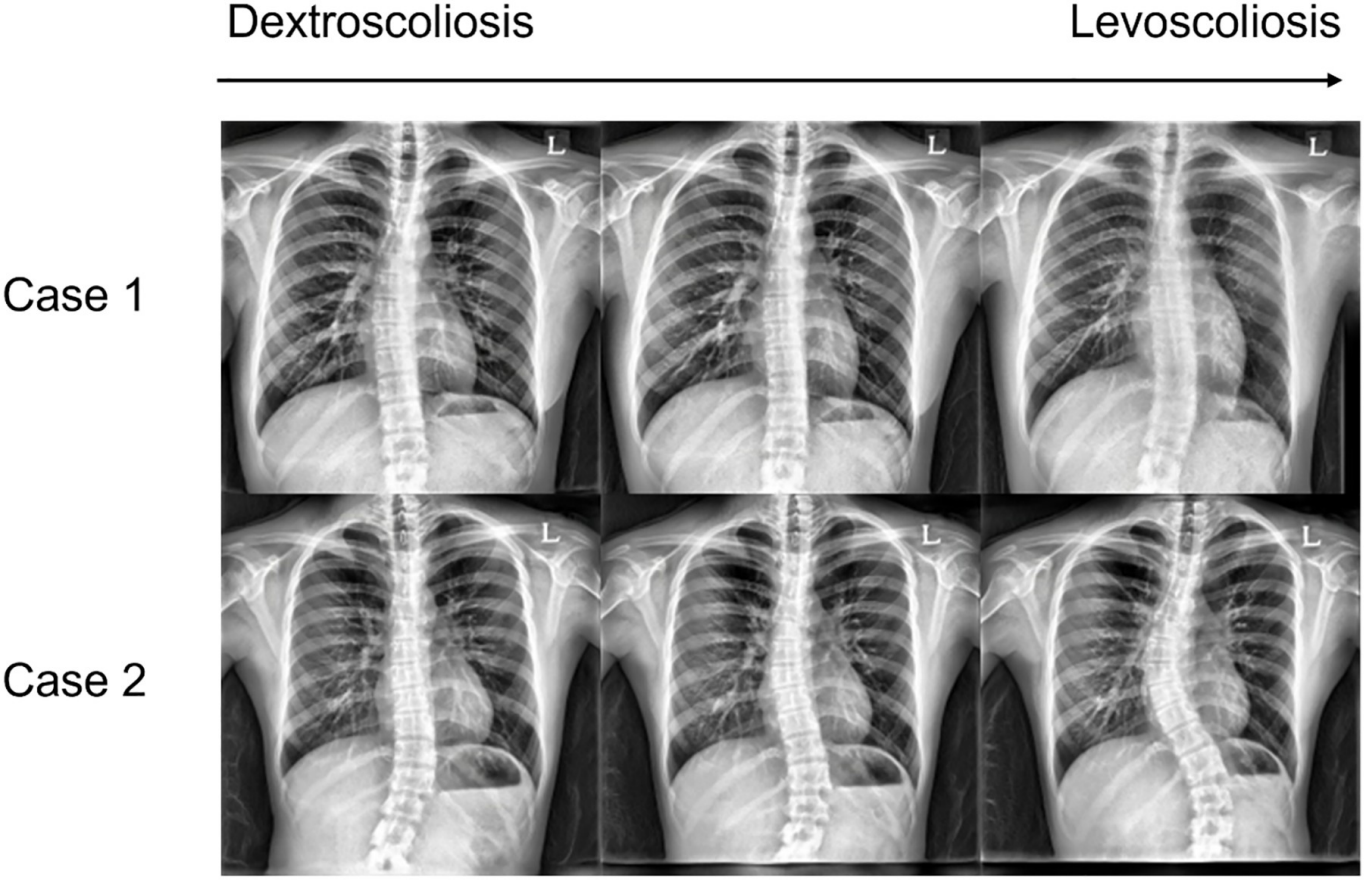
Two manipulated image examples. Note that scoliosis forms traverse between levoscoliosis and dextroscoliosis.

Furthermore, to demonstrate that even normal images are embedded in the latent space, we manipulated images using the “scoliosis direction” found by navigating the latent space in an unsupervised manner [25]. As a result, we confirmed normal images generated from vectors in the latent space. Fig 8 shows plausible normal image examples generated from image manipulation from scoliosis images.

## Conclusion

We developed a classifier for AIS through generative representation learning. Our model shows good AUROC under screening chest radiographs in both the internal and external datasets. Our model has learned the spectral severity of AIS, enabling it to generate normal images even when trained solely on scoliosis radiographs.

## Supporting information

S1 File (DOCX)
