# Supplementary material for: Screening of adolescent idiopathic scoliosis using generative adversarial network (GAN) inversion method in chest radiographs
Source: PLoS One. 2023 May 22;18(5):e0285489. doi: 10.1371/journal.pone.0285489 (PMC10202263; doi:10.1371/journal.pone.0285489)
Supplement: S1 File — (DOCX) [file pone.0285489.s001.docx]

**Supplementary materials**

***Training Configurations***

For training upstream GAN network, we utilized StyleGAN-ADA [1] architecture. We inherited implementation details from Pytorch [2] implementation provided by authors of [1]. The training data was preprocessed according to the method mentioned in *Section 3.1.2*. The augmentation in input data to the discriminator during styleGAN2-ADA training was carried out with maximal combinations of the provided pipeline. However, we excluded some augmentations that are not appropriate in medical deep learning, such as horizontal flip or cutout. For the loss function, we used non-saturating loss [3] with R1 regularization [4], utilizing 6.5536 as the coefficient value. Finally, we used ADAM [5] optimizer with a learning rate of 0.002. To evaluate the convergence of the upstream network, we used The Frechet inception distance (FID) [6] on the total training dataset. We selected epochs that showed the lowest FID value.

For feature extraction, we used a simple loss that minimizes the L2 norm of the given query image and the generated image, with a noise regularization term. Then, we iterated 1000 times to extract the final vector without weight update to the GAN generator.

For classifying the extracted vectors, binary cross-entropy was used as a loss to train the binary classifier. The ratio of normal and abnormal in the downstream training set was set to 1:1, and the number of training data in each dataset started from 32 and doubled up to 1024. The model was trained for 200 epochs with full batch learning and the learning rate was set to 0.001 in the Adam optimizer.

**References**

1. Karras T, Aittala M, Hellsten J, Laine S, Lehtinen J, Aila T. Training generative adversarial networks with limited data. arXiv preprint arXiv:200606676. 2020.

2. Paszke A, Gross S, Massa F, Lerer A, Bradbury J, Chanan G, et al. Pytorch: An imperative style, high-performance deep learning library. Advances in neural information processing systems. 2019;32:8026-37.

3. Goodfellow I, Pouget-Abadie J, Mirza M, Xu B, Warde-Farley D, Ozair S, et al. Generative adversarial nets. Advances in neural information processing systems. 2014;27.

4. Mescheder L, Geiger A, Nowozin S, editors. Which training methods for GANs do actually converge? International conference on machine learning; 2018: PMLR.

5. Kingma DP, Ba J. Adam: A method for stochastic optimization. arXiv preprint arXiv:14126980. 2014.

6. Heusel M, Ramsauer H, Unterthiner T, Nessler B, Hochreiter S. Gans trained by a two time-scale update rule converge to a local nash equilibrium. Advances in neural information processing systems. 2017;30.
